# Fibroblast activation protein α in tumor microenvironment: Recent progression and implications (Review)

**DOI:** 10.3892/mmr.2015.3197

**Published:** 2015-01-14

**Authors:** FUMING ZI, JINGSONG HE, DONGHUA HE, YI LI, LI YANG, ZHEN CAI

**Affiliations:** 1 Department of Hematology, Bone Marrow Transplantation Center, The First Affiliated Hospital, School of Medicine, Zhejiang University, Hangzhou, Zhejiang 310003, P.R. China; 2 Department of Hematology, The Second Affiliated Hospital, School of Medicine, Nanchang University, Nanchang, Jiangxi 330006, P.R. China

**Keywords:** cancer-associated fibroblasts, fibroblast activation protein α, tumor microenvironment

## Abstract

Accumulated evidence has demonstrated that the microenvironment of a given tumor is important in determining its drug resistance, tumorigenesis, progression and metastasis. These microenvironments, like tumor cells, are vital targets for cancer therapy. The cross-talk between tumor cells and cancer-associated fibroblasts (CAFs, alternatively termed activated fibroblasts) is crucial in regulating the drug resistance, tumorigenesis, neoplastic progression, angiogenesis, invasion and metastasis of a tumor. Fibroblast activation protein α (FAPα) is a transmembrane serine protease and is highly expressed on CAFs present in >90% of human epithelial neoplasms. FAPα activity, alongside that of gelatinase and type I collagenase, has become increasingly important in cancer therapy due to its effectiveness in modulating tumor behavior. In this review, recent progression in the knowledge of the role of FAPα in tumor microenvironments is discussed.

## 1. Introduction

Cancer tissue is a sophisticated construct of both malignant tumor cells and nonmalignant host stromal cells. In 1889, Paget (1) proposed a novel concept, the ‘seed and soil’ hypothesis, postulating that the congenial microenvironment (the ‘soil’) is prerequisite for the progression of tumor cells (the ‘seeds’). Tumor cells are disseminated throughout the body via the blood stream, but only in congenial ‘soil’ can metastases develop. In the past, cancer research primarily focused on neoplastic cells. This led to a rapid progression of knowledge pertaining to the genetic and epigenetic changes they undergo and elucidation of their signaling pathways in tumor cells (2,3). Despite the advancement of knowledge in the malignant transformation of tumor cells, existing therapies remain relatively ineffective for most types of cancer. Hertenstein *et al* (2), Sala-Torra *et al* (5) and Xiao *et al* (6), respectively, reported that leukemia patients suffered from donor cell leukemia (DCL) following allogeneic hematopoietic stem cell transplantation (allo-HSCT). In Xiao *et al’*s study (6), the patient in question as well as his donor-sister had the CCAAT enhancer binding protein α genetic abnormality, however, leukemia did not manifest in the patient’s sister. Other studies have also demonstrated that cells containing abnormal genetic changes only lead to tumor formation in a congenial microenvironment (7–10).

The results of the aforementioned studies revealed that genetic abnormality in tumor cells alone is not sufficient to produce cancer cells with malignant characteristics. The tumor microenvironment may be a necessity in the inception of malignant tumors and is increasingly being recognized to have a vital role in the progression of solid tumors and hematological malignancies (11–14). Cancer-associated fibroblasts (CAFs) are the most ubiquitous element of tumor stroma and are found in numerous types of cancer, including breast (15,16), NSCLC (17), colorectal (18–20), liver (21) and prostate cancer (22). The exact origin and specific markers of CAFs remain to be elucidated. Contemporary knowledge suggests that CAFs may be derived from: *i*) Local resident fibroblasts that undergo education by tumor cell-secreted cytokines; *ii*) bone marrow-derived mesenchymal stem cells (BMMSCs); *iii*) cancer cells undergoing epithelial-mesenchymal transition (EMT); *iv*) endothelial cells undergoing endothelial-to-mesenchymal transition (EndoMT); and *v*) other mechanisms (23–25). Fibroblast activation protein α (FAPα) is an important surface marker of CAFs. Aggregated data revealed that the elimination of FAPα led to stunted tumor growth and progression and stimulated the immune system to enhance the effects of tumor vaccination (26–30).

In the present review, the current knowledge regarding the role of FAPα in the interaction between cancer cells and the tumor microenvironment, as well as its biological and therapeutic implications, were summarized.

## 2. The discovery of FAPα

In 1986 and 1988, using the monoclonal antibody (mAb) F19, Rettig *et al* (31,32) identified a surface protein-F19 on the reactive stromal fibroblasts of epithelial cancers, most soft tissue sarcomas and granulation tissue of wound healing and certain fetal mesenchymal tissues, including fibroblasts in the dermis, perichondrium, renal capsule and peritoneum. Conversely, it was found that the stroma of benign epithelial tumors, normal and malignant epithelial cells, malignant hematopoietic cells, as well as normal stromal fibroblasts of the fetal kidney, colon, lung and cartilage and skeletal muscle were F19-negative (31). Subsequently, this mAb F19-identified protein was named fibroblast activation protein (FAP) (33–36). The human FAP, a cell surface protein, is comprised of Mr 95,000 (p95, FAPα) and Mr 105,000 (p105, FAPβ) subunits, which are conjugated by noncovalent, non-disulfide bonds. FAPβ is identical to T cell activation protein CD26 (also known as dipeptidyl peptidase 4, DPP 4) (35,37). Immunoblot experiments revealed that FAPα, but not FAPβ, carries the epitope defined by mAb F19 (33) and the F19 surface antigen was renamed as FAPα. In 1990, Aoyama and Chen (38) identified a dimeric 170 kDa membrane-bound gelatinase in the invadopodia of the aggressive malignant human melanoma cell line LOX. In 1994, this dimeric 170 kDa gelatinase was given the name ‘seprase’ (39). Subsequent cloning and sequence analysis of FAPα and seprase indicated that they were the identical transmembrane protease (40,41). In the present review, the term FAPα was used to denote this serine protease.

## 3. The structure of FAPα

**Figure 1:**
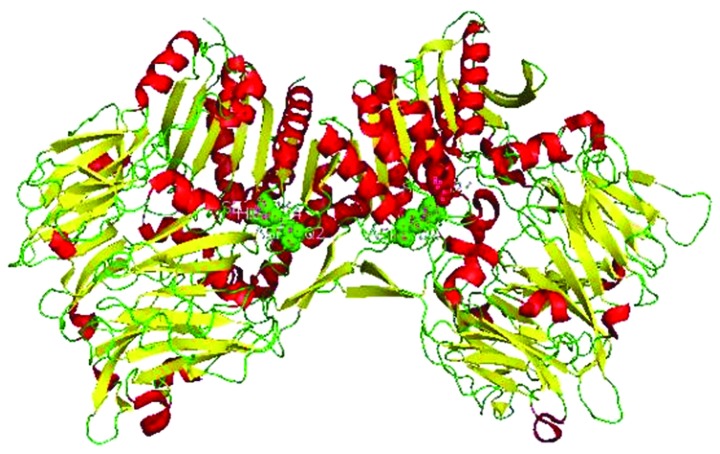
Ribbon diagram demonstrating the architecture of the fibroblast activation protein α dimer. Active amino acid residues Ser624, Asp702 and His734 are represented in sphere representations. The figure was generated using PyMOL (PDB ID 1Z68). Red, helix; yellow, β-sheet; green, loop and others.

FAPα, expressed in activated stromal fibroblasts and remodeling tissue, is a type II cell-surface-bound transmembrane glycoprotein with Mr 95,000. It consists of 760 amino acids, most of which possess a hydrolytic area exposed laterally of the plasmalemma. ~20 amino acids are anchored in the plasma membrane, and 6 amino acids are located in the cytoplasm (42). The conserved catalytic triad of FAPα is comprised of serine (S624), aspartate (D702) and histidine (H734) (42,43) (Fig. 1). FAPα is a member of the peptidase S9b family, a serine prolyl oligopeptidase subfamily, with post-prolyl peptidase activities able to cleave proteins and peptides following proline residues at the penultimate and P1 positions (44). In addition to FAPα (EC=3.4.21), this S9b serine peptidase family includes dipeptidyl peptidase 4 (DPP4, also termed CD26, which is identical to FAPβ, EC=3.4.14.5), dipeptidyl aminopeptidase-like protein 6 (also named DPPX or DPP6), DPP8 (EC=3.4.14.5), DPP9 (EC=3.4.14.5) and DPP10, and has been implicated in diabetes, cancer and inflammatory diseases (45–47) (additional information is available at: http://www.uniprot.org; http://enzyme.expasy.org). FAPα shares 48% amino acid sequence identity with DPP4 (35). FAPα and DPP4 are able to form homodimer FAPα/FAPα or heterodimer FAPα/DPP4 complexes to execute functions. The FAPα monomer is inactive, therefore dimerization is prerequisite for its catalytic function (43,48,49). FAPα and DPP4 are encoded by genes on human chromosomes 2q23 and 2q24.3, respectively (41,50). DPP8 and DPP9 are localized to chromosomes 15q22 and 19p13.3, respectively (51). DPP6 is encoded by a gene on human chromosome 7 (41,52) and DPP10 is encoded by a gene localized to chromosome 2 (2q12.3–2q14.2) (47). Murine FAPα shares 89% amino-acid-sequence identity with human FAPα (37). A promoter element of FAPα, early growth response 1 (EGR1), has been described (53).

## 4. Expression of FAPα in the tumor microenvironment and in benign diseases

Approximately 90% of reactive stromal fibroblasts of epithelial tumors, but not malignant tumor cells, overexpress FAPα (31,54). Immunohistochemical analysis using formalin-fixed and paraffin-embedded sections disclosed expression of FAPα in infiltrating ductal carcinomas (IDC) (55). The data indicated that the majority of stromal fibroblasts of epithelial tumors and certain malignant tumor cells are characterized by an overexpression of FAPα (Table I).

Further to overexpression in the cells and tissues mentioned above and summarized in Table I, FAPα is also expressed in certain benign diseases and normal tissues.

In 1999, Levy *et al* (56) examined 17 cirrhotic and eight normal liver samples by immunohistochemistry and RT-PCR. The results indicated that FAPα was mainly expressed in the hepatic stellate cells or perisinusoidal cells in periseptal regions of cirrhotic liver samples. Acharya *et al* (57) found that FAPα was expressed on fibroblast foci and in the fibrotic interstitium of patients with idiopathic pulmonary fibrosis (IPF), but was not expressed in normal or centriacinar emphysemal human lung tissue. Bauer *et al* (58) examined the FAPα-expression of fibroblast-like synoviocytes (FLSs) from patients with rheumatoid arthritis (RA) and osteoarthritis (OA) and found that FAPα expression was higher in FLSs from RA patients than in those from OA patients. Immunohistochemical analysis indicated that FAPα was forcefully expressed in the submucosa of Crohn’s disease (CD) strictures, but not in the submucosa of nonstrictured areas, ulcer (UC) submucosa or normal submucosa (59). FAPα expression was also observed in coronary atheromata, particularly in thin-cap atheromatas (60).

In normal tissue, cultured fibroblasts, but not resting fibroblasts, have a strong expression of FAPα. The cultural conditions may mimic the ‘wounds that do not heal’ state. Another important normal cell type that expresses FAPα is bone marrow mesenchymal stem cells (BMMSCs) (61,62). In the light of present knowledge, it is difficult to make a clear distinction between BMMSCs and fibroblasts. It appears that MSCs and fibroblasts share properties beyond those previously understood and that MSCs may in fact be fibroblasts’ new ‘clothes’ (63). Therefore, BMMSCs may be regarded as cultured fibroblasts. Table I lists FAPα-expressing tissues.

## 5. Factors driving the expression of FAPα

FAPα expression may be elevated under the influence of an altered tumor microenvironment or inflammation. *In vitro* FAPα expression was observed in fibroblasts and melanocytes cultured in fibroblast growth factor (FGF) and phorbol ester (33). Treatment of FB20 cells with human transforming growth factor-βl (TGF-β1), 12-*o*-tetradecanoyl phorbol-13-acetate (TPA), retinol or retinoic acid for 24–48 h increased FAPα expression in the cells (34). FAPα expression in CD strictured myofibroblasts under the stimulation of 10 ng/ml tumor necrosis factor α (TNF-α) or TGF-β1 for 48 h was significantly increased (59). TNF-α, produced by macrophages, was also able to induce FAPα expression in cultured human aortic smooth muscle cells (60). Further to the cytokines and chemical substances which induce FAPα expression, physical stimulants, including ultraviolet radiation, also induce upregulation of FAPα expression in fibroblasts, melanocytes and primary melanoma cells to facilitate invasion and migration of the cells (69).

## 6. Tumorigenic and anti-tumor functions of FAPα

HEK293 cells and MDA-MB-231 human mammary adenocarcinoma cells were transfected with FAPα cDNA to constitutively express FAPα, and subsequently xenografted into SCID mice. The transfected cells were more likely to develop subcutaneous tumors and demonstrated enhanced tumor growth (70,71) as well as increased microvessel density (71), compared with mock-transfected cells. Antibodies that neutralized FAPα attenuated the tumor growth rate (70). The human breast cancer cell lines MDA-MB-435 and MDA-MB-436, stably transfected with anti-sense oligonucleotides of FAPα, demonstrated slower proliferation than their FAPα-expressing counterparts in serum-free medium but not in serum-containing medium, indicating that breast cancer cells with high FAPα expression levels may be independent from exogenous serum factors for growth (72). Planting FAPα-silenced SKOV3 cells in a xenograft mouse model resulted in significantly decreased tumor growth (73). This is consistent with the observation that the elimination of FAPα-expressing cells led to stunted tumor growth and enhanced anti-tumor immune response in a mouse model (30). Radioimmunotherapy with novel internalizing antibody ESC11 delayed growth of established tumors and extended survival of mice (74). Mutation at the site of Ser^624^→Ala^624^ of FAPα resulted in ~100,000-fold decrease in DPP activity and attenuated tumor growth when HEK293 cells transfected with enzymatic mutant (S624A) FAPα were inoculated subcutaneously into a CB17-SCID mouse (27). FAPα was upregulated in bone marrow mesenchymal stem cells and osteoclasts when co-cultured with myeloma cells and supported myeloma cell survival (61). Inhibition of FAPα with PT-100 (Val-boro-Pro) influenced the expression of adhesion molecules in osteoclasts and reduced myeloma growth and bone disease (75). In a mouse model, inhibition of FAPα with PT-100 resulted in an antitumor effect implicating tumor-specific cytotoxic T lymphocytes, protection of immunological memory, augmented antitumor activity of antibody-increasing cytokines [interleukin (IL)-1, IL-6, interferon, granulocyte-colony stimulating factor] and chemokines (76). Taken together, these studies indicated that FAPα is a tumor promoter.

Tumor immunotherapy is important for eradicating tumors with minimal residual disease. Tumor-associated antigens are able to spontaneously elicit a CD8(+) T-cell response (77). However, the results of therapeutic vaccination with such antigens in inhibiting tumor growth have been relatively ineffective. This may be associated with the immunosuppressive effect of the stromal cells surrounding tumors. A study demonstrated that depleting FAPα-expressing cells in a transgenic mouse elicited antitumor immunity, and thus indicated that FAPα-expressing cells are an immune-suppressive component of the tumor microenvironment (30). This result is in accordance with evidence that an oral DNA vaccine targeting FAPα is able to suppress primary breast carcinoma growth and metastasis (28). This process may be associated with a shift in the immune microenvironment from expression of T helper cells (T_h_)2 to T_h_1 (78).

While numerous studies demonstrated that FAPα was a tumor suppressor, in 1993, Rettig *et al* (33) observed that FAPα expression in melanocytes was downregulated once they transformed into malignant cells and acquired tumorigenic potential. Analysis of human skin lesions, detected by immunohistochemical analysis, indicated that FAPα was expressed in only a fraction of melanocytic nevi and expression was scarce in both primary and metastatic melanoma lesions (79). By hybridizing normal fibroblasts with tumorigenic and nontumorigenic HeLa cells, Tsujimoto *et al* (80) identified FAPα as a potential inhibitor of tumorigenesis. All these observations were consistent with Brown *et al*’s (81) discovery that *Xenopus laevis* demonstrate a marked expression of FAPα whilst reabsorbing tadpole tails during amphibian metamorphosis. This indicated that FAPα was a pro-apoptotic factor involved in tissue remodeling. FAPα also enhanced apoptosis in the mouse B16 melanoma cell line independent of DPP4 and its enzymatic activity (82).

Recently, a study using transgenic mice revealed that FAPα(+) cells may have important functions in maintaining normal muscle mass and hematopoiesis, and their expression in normal tissues may have an important role in the paraneoplastic syndromes of cachexia and anemia (83). Niedermeyer *et al* (84) found that, i*n vivo,* homozygous FAPα-deficient mice generated from homologous recombination in the embryonic stem cell line R1 were fertile and exhibited no overt developmental defects or general changes in cancer susceptibility. Therefore, the function of FAPα may vary between tumor contexts and require further study.

FAPα not only has an important role in regulating tumor behavior, but also influences CAF behavior. Silencing FAPα with short interfering RNA transfected using a lentiviral vector inhibited growth and resulted in cell cycle arrest at the G_2_ and S phases of cancer-associated fibroblasts *in vitro* (73).

## 7. FAPα in tissue remodeling

Tissue remodeling is important in development, wound healing, chronic inflammation, fibrosis and cancer. It is understood that an active stroma is essential for cancer cell invasion and metastasis (85). Invasion and metastasis of malignant cancer cells requires the degradation of the extracellular matrix (ECM). FAPα displays DPP and gelatinolytic activity as proved by gelatin zymography and can cleave native ECM proteins, including collagen I, collagen IV, fibronectin, laminin and gelatin (38–40,49,86). These enzyme activities depend on the mutation at position Ser^624^, which abrogates the DPP and collagenase activity of FAPα (49). These enzymatic activities indicate that FAPα may have a prominent role in tumor invasion, metastasis and angiogenesis (86–88). Clinical observation revealed that the overexpression of FAPα by ductal carcinomas is congruent with the invasion and metastasis of infiltrating ductal carcinomas (IDC) of the breast (55). Using an *in vivo*-like three-dimensional matrix system, Lee *et al* (89) observed that FAPα remodeled the ECM and increased the invasive capability and metastasis of pancreatic tumors, mediated by β1-integrin/focal adhesion kinase.

In 1999, Levy *et al* (56) examined the biochemical activities of FAPα and found that FAPα exhibited gelatinase- and DPP-like activities. They concluded that FAPα may contribute to the hepatic stellate cell-induced ECM changes associated with cirrhosis. Wang *et al* (90) also found that human embryonic kidney 293T cells and hepatic stellate cell (HSC) line LX-2 overexpressing FAPα increased staurosporine streptomyces-induced cell apoptosis. However, FAPα-overexpression in these cells had contrasting effects on cell adhesion and migration, causing a reduction in that of kidney 293T cells and an increase in that of LX-2 cells. These nonenzymatic functions of FAPα may function in liver-tissue remodeling through enhancement of HSC cell adhesion, migration and apoptosis (90).

**Figure 2:**
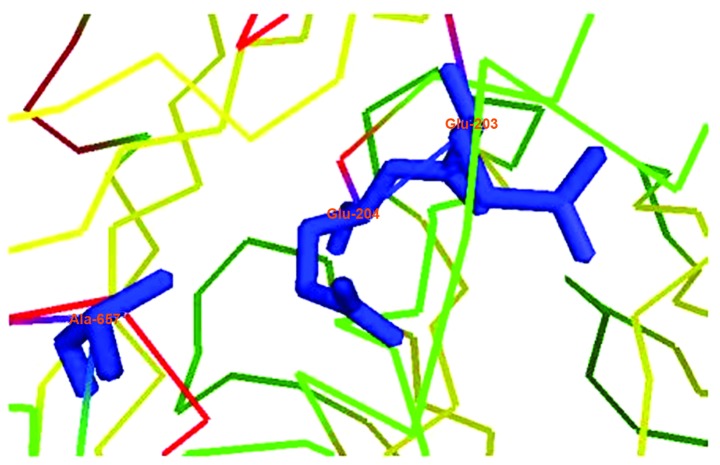
Glu motif of fibroblast activation protein α (Glu203-Glu204 and Ala657) with stick-representations. The figure was generated using PyMOL.

**Figure 3:**
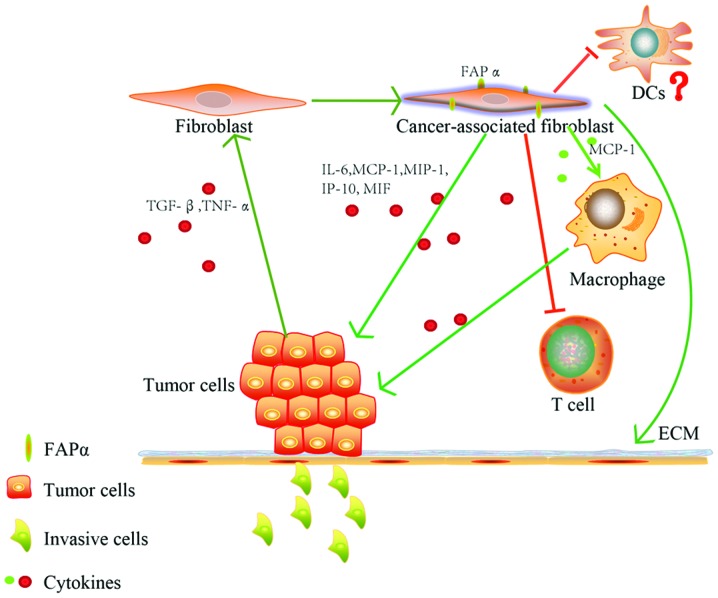
Intricate interaction of tumor cells with FAPα in tumor microenvironment. Tumor cells and its secreted cytokines (including TGF-β, TNF-α and SDF-1) educate resting fibroblasts to become activated fibroblasts with higher expression of FAPα. FAPα, through direct or indirect contact (cytokines, including IL-6, MCP-1, MIP and IL-1) supports tumor-cell survival. FAPα remodels the ECM and increases the invasive capability and metastasis of tumor cells. FAPα promotes cancer-associated fibroblasts to secrete MCP-1, mediating macrophage chemoattraction to the tumor microenvironment. The immune function of T cells was suppressed by FAPα, leading to immune anergy. FAPα, fibroblast activation protein alpha; TGF-β, transforming growth factor beta; TNF-α, tumor necrosis factor alpha; SDF-1, stromal cell-derived factor 1; IL, interleukin; MCP-1, monocyte chemoattractant protein-1; MIP, macrophage inflammatory protein 1; ECM, extracellular matrix; DCs, dendritic cells.

In addition to DPP activity, FAPα also demonstrates endopeptidase activity due to the presence of Ala^657^, which leads to decreased acidity in the active site of the FAPα Glu motif (E203–E204; Fig. 2) (43,49). Fig. 3 summarizes the intricate interaction of tumor cells with FAPα in the tumor microenvironment.

## 8. FAPα and its association with clinical prognosis

The role of FAPα is controversial as it remains associated with tumor promotion and inhibition; therefore, the clinical significance of FAPα expression requires further study. Using immunohistochemical analysis, Wikberg *et al* (90) found that FAPα was expressed by stromal fibroblasts in 85–90% of colorectal cancers and that increased FAPα expression in the cancer center, but not in the outlying regions, was associated with microsatellite instability, high CpG island methylator phenotype and poor prognosis. FAPα expression in pancreatic adenocarcinoma is associated with desmoplasia and a worse prognosis (64,92). Henry *et al* (93) reported that patients with colon cancer who had high levels of stromal FAPα expression were more likely to demonstrate progression of disease, latent occurrence or recurrence of metastases and poor prognosis. FAPα is also involved in tumor re-growth and recurrence and high FAPα expression is correlated with poor prognosis in rectal cancer following chemoradiotherapy (94). Conversely, Ariga *et al* (95), discovered that higher expression of FAPα in the mesenchyme of invasive ductal carcinoma of breast cancer is associated with longer overall and disease-free survival.

## 9. FAPα substrate cleavage

To date, numerous endogenous substrates of FAPα have remained to be elucidated. In 2004, Lee *et al* (96) discovered and purified a proteinase from human plasma, antiplasmin-cleaving enzyme (APCE), which is capable of cleaving the Pro12-Asn13 bond of Met-α2-antiplasmin (α2-AP) to yield Asn-α2-AP. Subsequently, this APCE was identified as a soluble form of FAPα (97). In addition to α2-AP, gelatin and collagen, further substrates have been identified. Recently, neuropeptide Y, B-type natriuretic peptide, peptide YY, incretins, substance P, glucagon-like peptide-1 and glucose-dependent insulinotropic peptide were identified as substrates of FAPα (98). A study indicated that α2-AP was not a robust substrate of FAPα *in vitro*, but a novel substrate, Spry2 (also called Sprouty2, a member of the Sprouty family) was identified (99).

## 10. Clinical applications of FAPα targeting

The general and abundant expression of FAPα in the stroma of tumors makes it a potential target for the diagnosis and therapy of numerous carcinomas. A phase I clinical study was executed and indicated that FAPα was highly expressed by reactive stromal fibroblasts in >95% of primary and metastatic tumors in patients with colorectal carcinomas (36). A phase I open-label study demonstrated that a humanized antibody (sibrotuzumab), directed against human FAPα expressed by advanced or metastatic FAPα-positive cancer, may be administered safely. However, the study did not indicate sibrotuzumab efficacy for the treatment of FAPα-positive cancer (100). In 2003, an early phase II trial of sibrotuzumab in patients with metastatic colorectal cancer revealed that progressive disease was evident in 15 out of 17 evaluable patients (101). T cells, engineered with FAPα-reactive chimeric antigen receptors and stimulated with FAPα or FAPα-expressing cell lines, degranulated and produced effector cytokines (102). However, adoptive transfer of FAPα-reactive T cells into mice infected with various tumors, mediated weak antitumor effects (102). FAPα-specific redirected T cells for the treatment of FAPα-positive malignant pleural mesothelioma are currently subject to clinical trials (103).

## 11. Conclusion

The tumor stroma has been increasingly recognized as a vital participant in tumorigenesis, drug-resistance, angiogenesis, invasion and metastasis in numerous types of cancer. FAPα is highly expressed in CAFs and is important in mediating their function. Ubiquitous expression by the majority of the stroma of epithelial tumors makes FAPα an ideal target for cancer therapy. Since the discovery of FAPα, it has been studied extensively. However, though a large amount of promising results were observed *in vitro*, clinical application of FAPα-targeting has thus far remained ineffective. In view of the complexity of its functions, FAPα requires further study.

## Figures and Tables

**Table I:** Tissue distribution of FAPα.

Antigen	Antigen-expressing cell types or tissues	References
F19	Cultured fibroblasts; granulation tissue; pancreatic islet (A) cells; fetal mesenchymal tissues (fibroblasts in the dermis, renal capsule, perichondrium, peritoneum); fibrosarcoma; malignant fibrous histiocytoma; leiomyosarcoma; osteosarcoma; hondrosarcoma; liposarcoma; synovial sarcoma; schwannoma, partial melanoma cell lines.	(32,33)
Seprase/FAPα	Melanoma cell line, infiltrating ductal carcinomas, pancreatic ductal adenocarcinoma and pancreatic cancer cell lines (SW1990, Miapaca-2, AsPC-1 and BxPC-3), cancer cells of colorectum, stomach and uterine cervix; glioma cells.	(38,55,64–68)
F19	Reactive mesenchyme of epithelial and nonepithelial tumors (colorectal, breast, ovarian and bladder tumors; lung cancer; mesothelioma; gastric, pancreatic, endometrial and neuroendocrine cancers; melanoma;lymphoma).	(36,54)
F19	Hepatic stellate cells of cirrhotic liver.	(56)
FAPα	Bone marrow-derived mesenchymal stem cells, osteoclasts, vascular endothelial cells, adipocytes.	(61,62)
FAPα	Fibroblast foci and fibrotic interstitium of idiopathic pulmonary fibrosis.	(57)
F19	Fibroblast-like synoviocytes of rheumatoid arthritis and osteoarthritis.	(58)
FAPα	Submucosa of Crohn’s disease strictures; atherosclerotic plaques.	(59,60)

FAPα, fibroblast activation protein α.
